# Risk factors for dehiscence in alveolar ridge augmentation using patient-specific titanium mesh: a retrospective analysis

**DOI:** 10.1186/s40729-025-00623-9

**Published:** 2025-06-05

**Authors:** Sebahat Kaya, Bhárbara Marinho Barcellos, Shengchi Fan, Adriano Azaripour, Christian Walter, Amely Hartmann, Keyvan Sagheb

**Affiliations:** 1 Department of Oral and Maxillofacial Surgery, Plastic Surgery, University Medical Center, Johannes Gutenberg, University, 55131 Mainz, Germany; 2 Hospital for Rehabilitation of Craniofacial Anomalies, University of São Paulo, 17.012-900, Bauru, São Paulo, Brazil; 3 Departmet of Dentistry, Oral Surgery and Implantology, Faculty of Medicine and Health Sciences, University of Barcelona, 08907 Barcelona, Spain

**Keywords:** Augmentation, Titanium meshes, Dehiscence, Guided bone regeneration, Dental implant

## Abstract

**Purpose:**

This retrospective study aimed to evaluate the incidence of dehiscence following bone augmentation with patient-specific titanium meshes and to identify factors associated with its occurrence.

**Material and methods:**

Patients who underwent bone grafting with patient-specific titanium mesh between December 2014 and October 2021 were included. The primary outcome was the occurrence of dehiscence. The occurrence of dehiscences was recorded during the following time phases, enabling the determination of whether dehiscences occur early (< 2 weeks), in the mid-term (2–9 weeks), or later in the healing phase (> 9 weeks).

**Results:**

A total of 78 patients undergoing 85 titanium mesh augmentations were included, with a mean follow-up period of 1.2 years. Dehiscence occurred in 33 meshes (38.8%), with 51.5% of these events arising during the early healing phase. In no case was premature removal of the titanium mesh required due to dehiscence. A statistically significant association was observed between dehiscence and both smoking behavior (*p* < 0.001) and the anatomical location of the maxillary defect (*p* = 0.029). No significant associations were found between dehiscence and gender (*p* = 0.160), periodontitis (*p* = 0.512), gingival phenotype (*p* = 0.495), defect type (*p* = 0.490), augmented bone volume (*p* = 0.373), or incision type (*p* = 0.354). Logistic regression analysis further identified smoking (odds ratio: 7.07; 95% CI: 2.19–22.80) and maxillary defect alveolar (odds ratio: 11.86; 95% CI: 0.34–4.60) as significant predictors of dehiscence.

**Conclusion:**

Dehiscence following customized titanium mesh augmentation was significantly associated with smoking and the location of the maxillary defect, underscoring the importance of early detection and timely intervention.

**Graphical Abstract:**

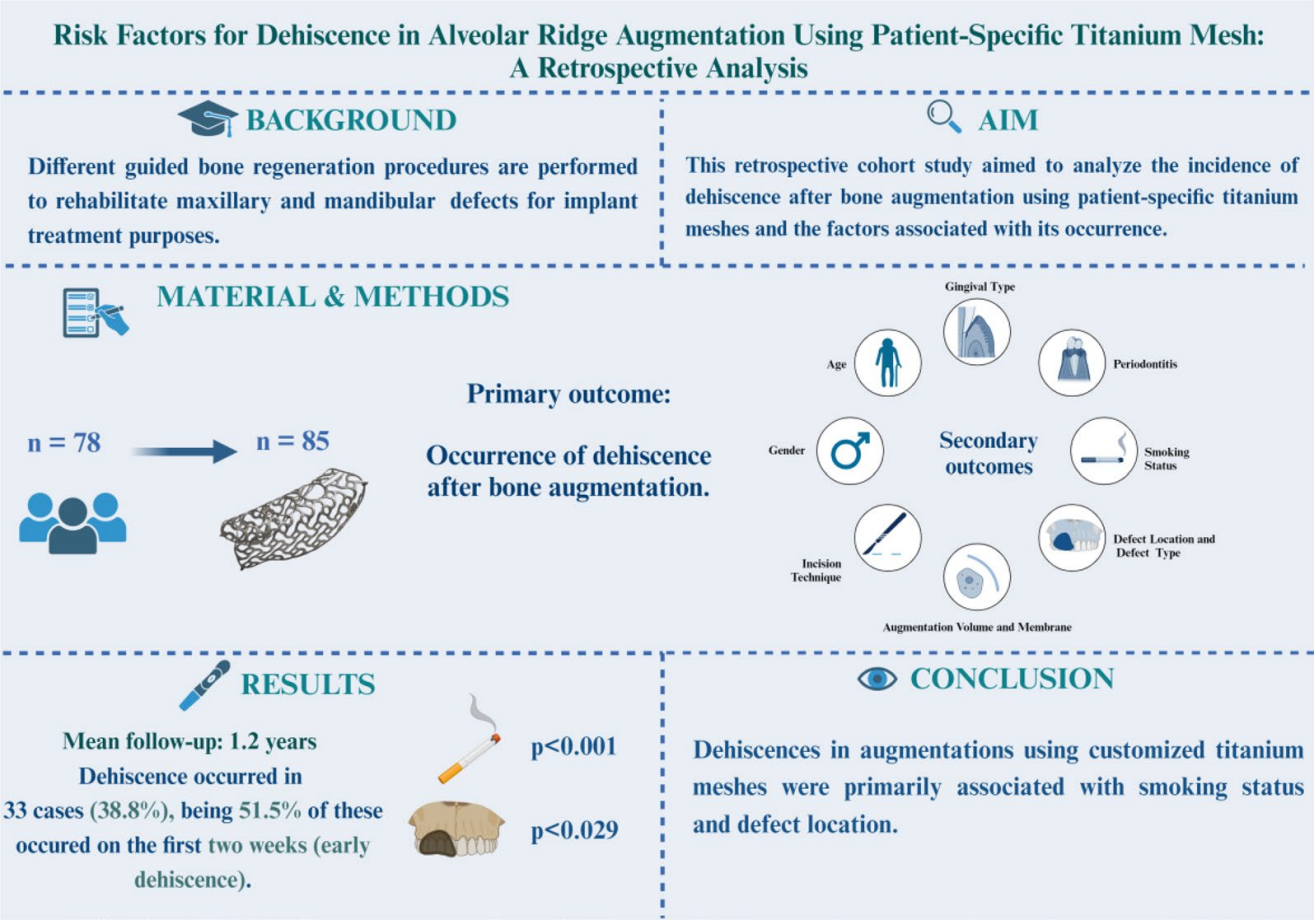

## Introduction

Different bone augmentation techniques have been introduced and applied for alveolar atrophy and defects in the maxilla and mandible [1]. Among the various bone augmentation techniques, guided bone regeneration (GBR) procedures are performed according to the extent of the defect and the type of defect using autogenous bone particles or bone substitutes in combination with resorbable or non-resorbable membranes [2].

The titanium mesh has been utilized as a non-resorbable barrier for the treatment of vertical and horizontal bone defects with a high success rate [3]. However, complications such as tissue dehiscence and graft failure have been reported, attributed mainly to mesh rigidity or sharp edges [4]. Additionally, surgical site closure is more challenging due to the considerable volume of bone substitutes and the rigidity of titanium barrier, leading to increased tension on the soft tissue.

Advances in digital workflows have enabled individualized fabrication of titanium meshes, potentially reducing tissue trauma and exposure [5]. However, tissue dehiscence remains a significant complication compared to other augmentation methods [6]. This study aimed to evaluate the incidence of soft tissue dehiscence following augmentation with customized titanium meshes and to identify associated risk factors.

## Material and methods

This retrospective cohort study was designed to analyze patients with defective alveolar ridges requiring bone augmentation prior to implant treatment. The study included 85 bone augmentation procedures using customized titanium meshes (Yxoss meshes, ReOss, Filderstadt, Germany) performed between December 2014 and October 2021 at the Department of Oral and Maxillofacial Surgery at the University Medical Center of Mainz, Germany. The manuscript was prepared according to the Strengthening the Reporting of Observational Studies in Epidemiology (STROBE guidelines). The ethics committee of the University Medical Center, Mainz, Germany approved the ethical approval (2024–17628).

### Patients and study design

The inclusion criteria were as follows: (1) Patients with bone defects requiring 4–6 mm of alveolar ridge augmentation horizontally with or without vertical augmentation; (2) Patients who had completed bone augmentation with patient-specific titanium mesh and had at least a 1-year follow-up; and (3) Patients in good physical health and able to maintain oral hygiene. Patients with smoking status were educated on the possible negative impact of smoking on the success of treatment. Exclusion criteria included contraindications for surgical procedures, such as uncontrolled systemic conditions, uncontrolled periodontal disease, heavy smoking (more than 10 cigarettes per day), excessive alcohol consumption, and poor oral hygiene. The endpoint of this study was to evaluate the dehiscence rate in titanium mesh augmentation and to identify the factors that might influence its occurrence. The endpoint of this study was to evaluate the dehiscence rate in titanium mesh augmentation and to identify the factors that might influence its occurrence.

Patient-specific data:

- Age
- Gender
- Health status
- smoking habit (less than 10 cigarettes per day)
- periodontitis [7]
- Gingival phenotype [8]
- thin (probe visible, ≤ 1 mm)
- thick (probe not visible, > 1 mm)
- Defect location (Maxilla or Mandible)
- Defect type defined (horizontal, vertical and combined)

Operation-specific factors:

- Augmentation Volume (cm^3^)
- Incision type
- Membrane

Postoperative data:

Post-operative data were also documented in the form of operative reports and digitally stored on System Applications and Products in Data Processing (SAP^®^) with corresponding operation-relevant information:

- Dehiscence occurrence
- Follow-up period

### Mesh design

All the patients received a CBCT scanning for diagnosis purposes and treatment planning. First, virtual implant planning was applied to verify the required volume of bone surrounding the implanted sites [8]. The planning of implant position gave information on the bone volume for augmentation.

**Fig. 1:**
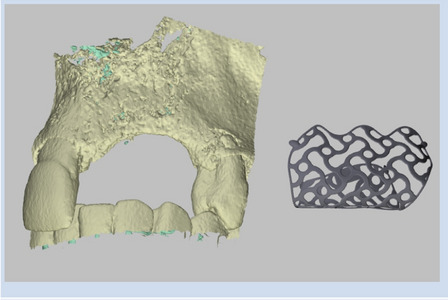
The patient lost the left maxillary first incisor (21), second incisor (22), and canine (23), resulting in horizontal and vertical bone defects. Based on the bone volume requirements from the implant planning, a 3D personalized titanium mesh was designed using specialized software

A three-dimensional (3D) model of the patient-specific bone defect was then created on the digital volume tomography (DVT). The grid is then produced on the 3D visualization using reconstruction software (Reverse Engineering Software) (Fig. 1) [5]. Thus, by segmenting bone structures from the model, the design of mesh structure and the pins for fixation were individually created according to the planned implant positions and necessary augmentation volume. The standard thickness of titanium meshes was 0.3 mm.

### Staged operation

**Fig. 2:**
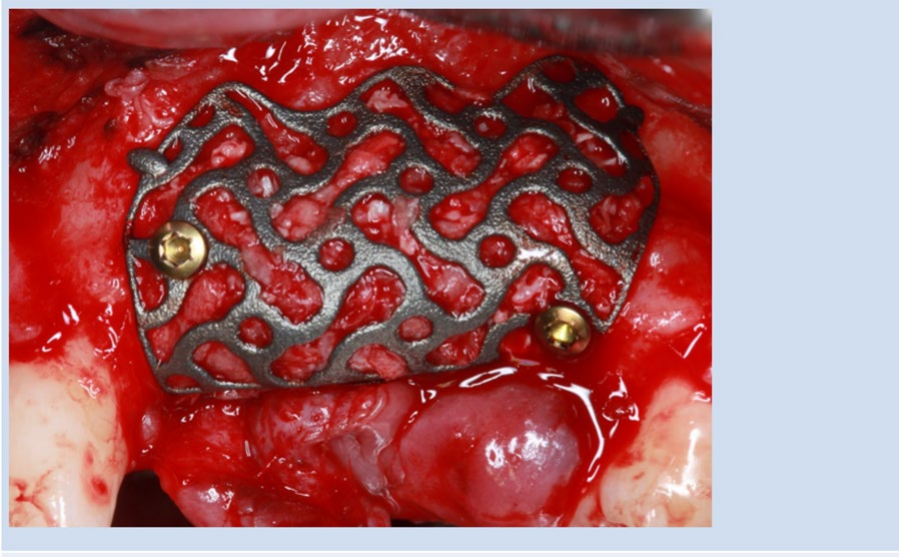
Autogenous bone harvested from the patient was mixed with deproteinized bovine bone mineral and then placed into the titanium mesh. Self-tapping titanium screws were used to fix the titanium mesh in the planned position

All patients underwent surgery under local anesthesia, and the choice of incision type (crestal or poncho) was determined individually based on clinical considerations. The surgical procedures, performed by an experienced surgeon (K,S), involved incision, flap elevation, and exposure of the defect prior to place and fixation of the mesh. After preparation of the defect area, the bone surface was contoured to optimize adaptation, ensuring adequate conditions for mesh placement. The mesh was positioned without further modification, maintaining a minimum distance of 1.5 mm from adjacent teeth and nerve structures. A combination of autologous bone and bone substitute materials, or bone substitute alone, was packed within the mesh. Following verification of correct mesh placement, osteosynthesis screws were inserted at the predetermined locations (Fig. 2). A resorbable collagen membrane was then applied over the mesh, and the surgical site was closed using tension-free sutures.

### Follow-up

All patients were called back for follow-up appointments at 1 week, 2 weeks, 1 month, 6 months, and 1 year postoperatively, or as needed. The occurrence of dehiscence was observed from the day of augmentation until the removal of the mesh. Then, it was recorded during the following time phases, enabling the determination of whether dehiscences occur early (< 2 weeks), in the mid-term (2–9 weeks), or later in the healing phase (> 9 weeks).

### Statistical analysis

The combined data and parameters in the Excel spreadsheet were statistically analyzed using the statistical program IBM SPSS version 27 (IBM, Armonk, NY, USA).

Descriptive analysis was conducted on various frequency distributions concerning patient-specific factors and operation-specific factors. Kaplan and Meier’s (1958) statistical analysis was employed to evaluate the rate of dehiscence-free healing based on smoking and the time-point of dehiscence occurrence. The associations between various patient-specific factors (gingival types, medical status factors, defect types) and operation-specific factors (incision techniques) with the occurrence of dehiscence were analyzed using the Pearson chi-square test or Fisher’s exact test. The significance level was set at 5% (p < 0.05). Logistic regression analysis was further conducted to evaluate the impact of predictors such as smoking, gender, age, defect location, and defect type on the likelihood of dehiscence occurrence. The results of the logistic regression analysis, including odds ratios and 95% confidence intervals, were used to assess the strength and significance of these associations, with a p-value of < 0.05 indicating statistical significance.

## Results

### Patient, and surgery characteristics

A total of 78 patients (54 female, 24 male) were included in the present study with a mean follow-up of 1.2 ± 0.8 years (ranging from 5 months to 2.1 years). The mean age was 44.7 ± 6.4 years. A total of 85 augmentation procedures were performed and analyzed in 78 patients, including 7 patients (5 women and 2 men) who underwent simultaneous augmentation at two separate sites. The information regarding the patient and clinical characteristics is summarized in Table 1.

**Table 1:** Patient and clinical characteristics

Characteristics		Number (%)
Total patients		78 (100)
Gender	Men	24 (30.7)
	Women	54 (69.3)
Age (Mean ± SD)	Men	46 ± 14
	Women	44 ± 17
Smoking	Yes	32 (37.6)
	No	46 (62.4)
Periodontitis	Yes	40 (47.1)
	No	38 (52.9)
Augmented sites		85 (100)
Gingival phenotype	Thin	45 (52.9)
	Thick	40 (47.1)
Defect location	Maxilla	34 (40.0)
	Mandible	51 (60.0)
Type of defect	Horizontal	16 (18.8)
	Vertical + Horizontal	69 (81.2)
Augmentation material	Autologous bone	4 (4.7)
	Autologous bone + bone substitute material	81 (95.3)
Incision types	Crestal	65 (76.5)
	Poncho	20 (23.5)

### Wound dehiscence and mesh exposure

**Fig. 3:**
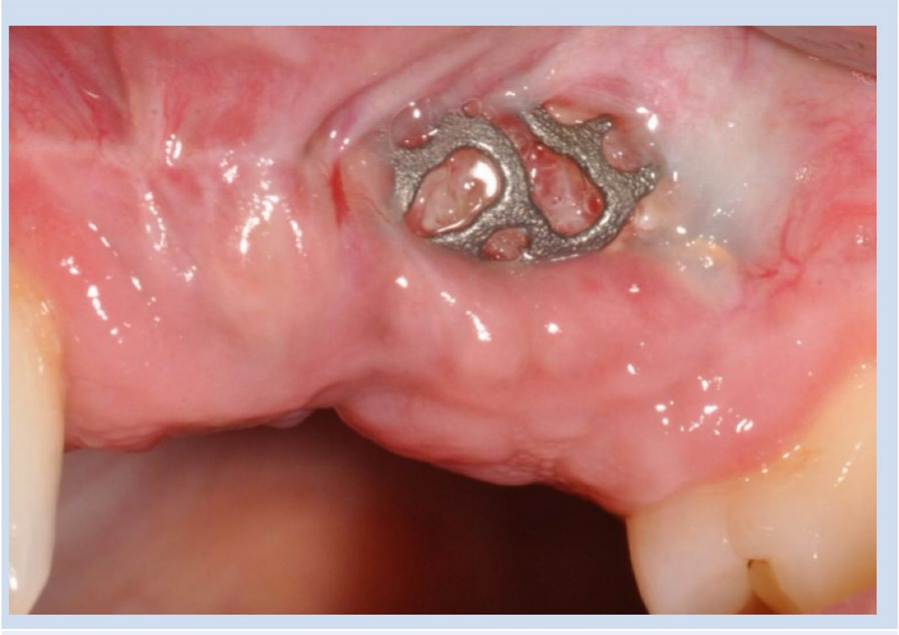
Upon operation after 6 weeks, an exposure of the titanium mesh measuring 10 × 15 mm was observed in the vestibular mucosa

**Fig. 4:**
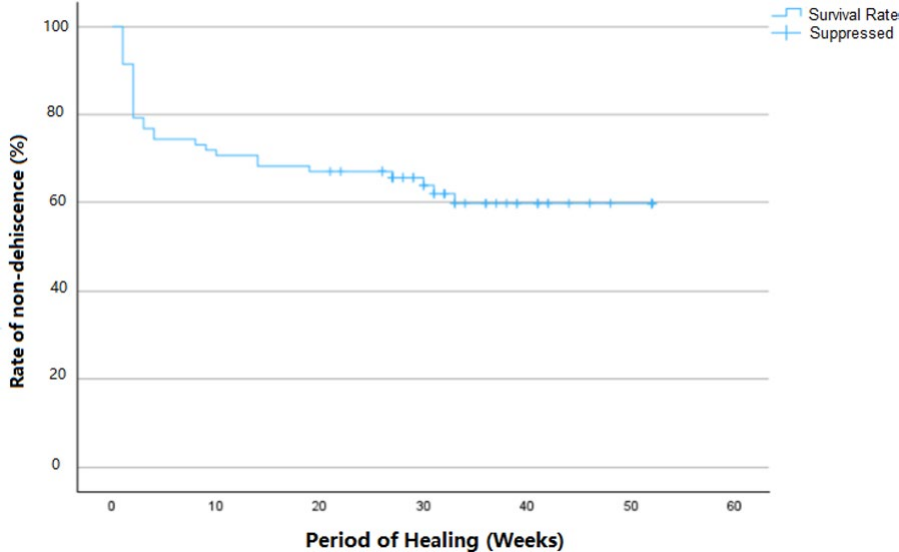
The highest frequency of dehiscence was observed within the first 2 weeks (51.5%), followed by the mid-term period (18.2%), and the late period (30.3%)

**Fig. 5:**
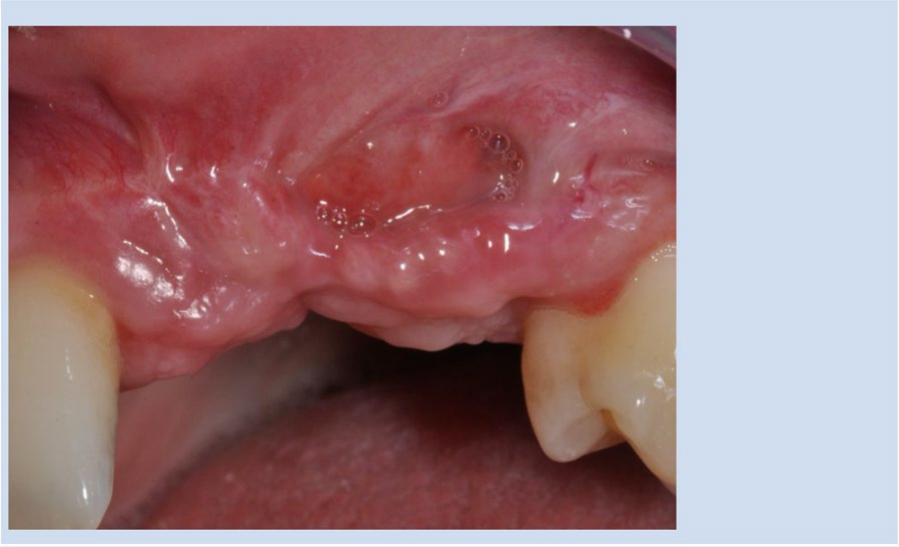
The patient was prescribed a chlorhexidine mouthwash rinse and instructed to maintain meticulous oral hygiene. After two weeks of healing, the dehiscence was completely resolved with soft tissue coverage

Dehiscence was observed in 33 cases (38.8%), with 51.5% of these observed within the first two weeks, indicating an early onset. Mid-term dehiscences were the least frequently observed (Fig. 3), whereas late-onset dehiscences accounted for 30.3% of cases (Fig. 4). The examination of gender-specific occurrences revealed that women developed dehiscences less frequently, with a rate of just under 34%, compared to men with a rate of 50%. However, there was no significant association between genders and the occurrence of dehiscence (p = 0.160). Upon observing dehiscence, patients were treated with a chlorhexidine mouthwash rinse and instructed to maintain oral hygiene. In none of the cases was premature removal of the titanium mesh necessary following dehiscence and exposure (Fig. 5).

### Risk factors for dehiscence analysis

Overall, 37.6% of the patients were smokers, with a significantly larger proportion of male patients compared to female patients (28.8%). Of the patients with periodontitis, 42.4% were women and 57.6% were men.

**Fig. 6:**
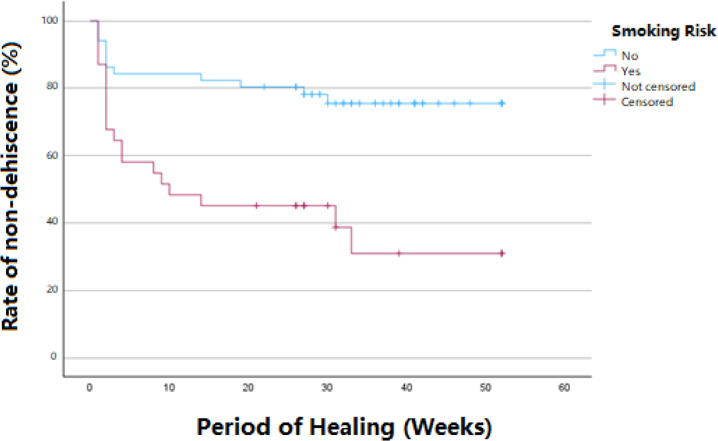
Dehiscences occur earlier and with a greater overall frequency in the group of smokers compared to non-smokers (p < 0.001)

Among smoking patients, the occurrence of dehiscences was statistically significantly higher (62.5%) compared to non-smokers (24.5%) (p < 0.001). Dehiscence in the smoking group occurred predominantly in the early period (Fig. 6).

In total, 42.5% of the patients with periodontitis presented dehiscence. However, there is no significant correlation between periodontitis and dehiscence occurrence (p = 0.512). Dehiscence in periodontitis group occurred predominantly in the early period.

### Gingival phenotype

Gingival phenotype was assessed using the transparency method with a periodontal probe and categorized as thin (probe visible, ≤ 1 mm) or thick (probe not visible, > 1 mm) [9]. 52.9% of patients presented with a thin gingival type, which was more prevalent among women (34.6%). Conversely, a thick gingival type predominated among male patients (65.4%).

Regarding dehiscence occurrence, 42.2% with a thin gingival type developed dehiscence, while only 35% with a thick gingival phenotype experienced the complication. However, the influence of gingival type was not statistically significant (p = 0.495).

### Defect location and type

Of the augmented defects, 40% were located in the maxilla and 60% in the mandible. Dehiscence was observed in 52.9% of the maxillary augmentations compared to 29.4% in the mandibular sites. The difference in the occurrence of dehiscence between maxillary and mandibular sites was statistically significant (p = 0.029).

As for the type of defect, 18.8% were horizontal, while 81.2% exhibited a vertical + horizontal defect. The dehiscence rate for vertical + horizontal defects was 40.6%, approximately 10% higher than the rate for horizontal defects (31.3%). However, the association between the defect type and dehiscence was not statistically significant (p = 0.490).

Regarding the period of occurrence, early dehiscences were more frequent in the maxilla (72.2%). The occurrence of late (16.7%) and mid-term (11.1%) dehiscences constituted smaller proportions. In the mandible, late dehiscences were most common (46.6%). With both early (26.7%) and mid-term (26.7%) dehiscences occurring with equal frequency.

### Augmented volume (cm^3^)

The volume of bone augmentation was individually calculated for each patient based on defect size, ranging from 0.22 to 8.51 cm^3^. Patients who developed dehiscence had, on average, a larger augmented volume (1.96 ± 1.93 cm^3^) than those without dehiscence (1.39 ± 1.01 cm^3^); however, this difference did not reach statistical significance (p = 0.373).

### Incision type and membranes

The crestal incision was the most frequently applied (76.5%). Among procedures using crestal incision, dehiscence occurred in 41.5%; however, this association was not statistically significant (p = 0.354). Resorbable membranes were used in 97.6% of the procedures. The Bio-Gide^®^ membrane (Geistlich, Baden-Baden, Germany) was the most frequently used (85.9%). Due to the small number of cases without membrane application (control group), it was not possible to analyze the correlation between membrane usage and the occurrence of dehiscence.”

### Regression analysis

The binomial logistic regression model demonstrated a moderate fit. Smoking remained the strongest predictor of dehiscence (p = 0.001), with smokers having an increased risk compared to non-smokers (95% CI: 2.19–22.80). Additionally, the defect location (mandible or maxilla) in which the procedure was performed was significantly associated with dehiscence (p = 0.023), suggesting a higher likelihood of occurrence in the maxilla. Conversely, age (p = 0.128), gender (p = 0.740), and defect type (p = 0.646) were not significantly associated with the outcome in the multivariate model (Table 2).

**Table 2:** Predictors of dehiscence: logistic regression analysis with odds ratios and confidence intervals

					Confidence interval 95%	
Preditor	Estimate	Standard Error	p-value	Odds ratio	Lower limit	Higher limit
Gender	0.1801	0.5428	0.740	1.1973	0.41321	3.469
Age	− 0.0263	0.0173	0.128	0.9741	0.94159	1.008
Smoke	1.9560	0.5973	0.001	7.0709	2.19295	22.799
Defect location	2.473	1.087	0.023	11.86306	0.343	4.604
Defect type	0.1503	0.3278	0.646	1.1622	0.61137	2.209

## Discussion

The most common complication of bone augmentation using titanium meshes is mesh exposure through dehiscence. Various studies have explored and investigated different therapeutic approaches both before and after the occurrence of dehiscence, aiming to prevent it as much as possible or manage further complications [6, 10, 11]. In this study, dehiscence in augmentations using customized titanium meshes was primarily associated with smoking and defect location in the maxilla.

The results of the dehiscence rate determined in this study were 38.8%, with 33 out of 85 augmented sites developing mesh exposure. These results are consistent with a systematic review conducted by Gue et al., in which three possible factors (employment of 3D-customized titanium mesh; type of bone graft material; use of resorbable membrane) were considered the possible causes of dehiscence occurrence and the reported dehiscence rates ranged from 17.9% to 34.4% [12].

The occurrence was correlated with patient characteristics, specifically smoking behavior (p < 0.001) and the location of the maxillary defect (p = 0.029). Early dehiscences occurred most frequently in the maxilla (72.2%), followed by late (16.7%) and mid-term (11.1%) dehiscences. Meanwhile, late dehiscences predominantly occurred in the mandible (46.7%), followed by early (26.7%) and mid-term (26.7%) dehiscences. This factor is important to point out, as the distinction regarding the timing of mesh exposure within the first few weeks appears to affect bone healing compared to later occurrences [13]. If an early mucosal dehiscence becomes infected, it poses a relatively high risk to overall success [13].

Untreated infected dehiscence might lead to a partial loss of the augmentation [13, 14]. In cases of dehiscence due to local infection, the protocol treatment includes systemic antibiotic therapy for a period of 10 days, wound cleansing with chlorhexidine gel, and clinical observation [13]. This protocol is also indicated for the management of titanium mesh exposure [6]. Although removal of the titanium mesh was not indicated in these cases, the wounds healed by secondary intention and required careful monitoring due to the fragility of the tissue; unnecessary suturing should be avoided.

Various phases of wound healing are disrupted by harmful substances in nicotine, leading to unavoidable wound-healing disorders [15]. In the evaluated cases, a substantial proportion of patients exhibited risk factors including periodontitis and/or smoking. Notably, nearly half of these patients experienced dehiscence, with a higher incidence observed among smokers during the early healing phases compared to non-smokers. This correlation is emphasized because the negative effects of smoking on cell function during the wound-healing process are well documented [15]. The results from the logistic regression analysis further emphasized the significant role of smoking as a predictor of dehiscence, with smokers showing a notably higher odds ratio (7.07) and a statistically significant association (p = 0.001), which supports the well-documented negative impact of smoking on wound healing. However, this study found no significant correlation between periodontitis and dehiscence occurrence, findings corroborated by several other studies that also failed to demonstrate any significant influence of periodontitis on dehiscence rates [11, 16].

No significant correlation was found between gingival phenotype and the occurrence of dehiscence (p = 0.495). However, the slightly higher dehiscence rate observed in patients with a thin phenotype may be attributed to reduced stability and a higher risk of tearing during the titanium meshe placement and wound closure, as also described in previous study [11]. The literature also suggests that a thin phenotype type is significantly more challenging to handle intraoperatively compared to a thick one. Larger procedures involving significant alveolar defects may pose a higher risk for dehiscence due to prior manipulation and subsequent weakening or scarring of the soft tissue [16].

Regarding the location, a total of 34 out of 85 augmentations (40%) were performed in the maxilla, while the remaining 51 procedures (60%) were conducted in the mandible. The dehiscence rate in the maxilla was 52.9% (n = 18), significantly higher than the rate in the mandible, which was 29.4% (n = 15), showing a significant correlation (p = 0.029). Similarly, Sagheb et al. found a significantly higher dehiscence rate in the maxilla (66.7%) compared to the mandible (8.3%) (p = 0.009) [6]. Interestingly, the jaw in which the procedure was performed also emerged as a significant predictor in the logistic regression model (p = 0.023), suggesting that anatomical differences between the maxilla and mandible may influence the likelihood of dehiscence.

Several studies have investigated possible correlations between defect sites, mesh size, and the occurrence of mesh exposures. However, specific classifications of defect location did not significantly influence the dehiscence rate. In contrast, Uehara et al. found a significant correlation between the size of the defect to be augmented and the success rate of bone augmentation [17].

In this study, resorbable membranes were used in 97.6% of the procedures. In only two out of 85 augmentations, no membrane was used, and these patients did not develop dehiscence. As there was a limited control group, no definitive conclusion can be drawn regarding the correlation between the use of a membrane and the occurrence of dehiscence. Nevertheless, some authors recommend the use of resorbable membranes to avoid the risk of early dehiscence [18]. In this context, Cucchi et al. proved healing complication rates of about 33% in bone augmentation using titanium mesh without membrane, while the value for using titanium mesh with a combined application of mesh and resorbable membrane was about 13%. Although these values do not show statistically significant differences, these results indicate that the use of the membrane is favorable [10].

A statistical analysis of the relationship between augmentation material and dehiscence rate was not conducted due to the expected low statistical power. When considering the influence of different augmentation materials on the dehiscence rate, Her et al. found no significant difference in the use of different materials [19]. In contrast, Carini et al. observed a lower dehiscence rate, increased bone gain, and decreased bone resorption with the use of autologous bone [20].

Another factor that might be related to dehiscence is the incision type. Sagheb et al. also considered the influence of the incision technique on the dehiscence rate. Crestal incisions had a higher exposure rate of 45.5% compared to the modified Poncho incision technique, which had an exposure rate of 20%. All mesh exposures were located in the area of the previously made incision. However, this difference was not statistically significant (p = 0.221).

The limitations of the present study include the absence of a control group to compare dehiscence rates with and without membrane use, discrepancies in grafted bone volume, and the lack of standardization in the types of bone grafts used in different surgeries. Prospective controlled studies are needed to further investigate the relationship between membrane use, bone augmentation materials, and the occurrence of dehiscence.

## Conclusion

Within the constraints of this retrospective study, it was found that dehiscence in augmentations using customized titanium meshes is primarily associated with smoking status and defect location in the maxilla. Early detection and prompt management of dehiscence are essential to optimize clinical outcomes.

## Data Availability

No datasets were generated or analysed during the current study.

## Abbreviations

GBR: Guided bone regeneration
CBCT: Cone beam computed tomography
DVT: Digital volume tomography
AB: Autologous bone
SAP: System applications and products in data processing
